# Reversal of Multidrug Resistance by Apolipoprotein A1-Modified Doxorubicin Liposome for Breast Cancer Treatment

**DOI:** 10.3390/molecules26051280

**Published:** 2021-02-26

**Authors:** Duopeng An, Xiaochen Yu, Lijing Jiang, Rui Wang, Peng He, Nanye Chen, Xiaohan Guo, Xiang Li, Meiqing Feng

**Affiliations:** 1 Minhang Hospital & School of Pharmacy, Department of Biological Medicines Shanghai Engineering Research Center of Immunotherapeutics, Fudan University, Shanghai 201023, China; 18111030046@fudan.edu.cn (D.A.); 16111030014@fudan.edu.cn (X.Y.); lijing_jiang@fudan.edu.cn (L.J.); 20211030037@fudan.edu.cn (R.W.); 20111030068@fudan.edu.cn (P.H.); 17211030048@fudan.edu.cn (N.C.); 19211030037@fudan.edu.cn (X.G.); 2 Department of Biological Medicines & Shanghai Engineering Research Center of Immunotherapeutics, Fudan University School of Pharmacy, Shanghai 201203, China

**Keywords:** Apolipoprotein A1, doxorubicin, multidrug resistance, breast cancer, MCF-7/ADR

## Abstract

Multidrug resistance (MDR) remains a major problem in cancer therapy and is characterized by the overexpression of p-glycoprotein (P-gp) efflux pump, upregulation of anti-apoptotic proteins or downregulation of pro-apoptotic proteins. In this study, an Apolipoprotein A1 (ApoA1)-modified cationic liposome containing a synthetic cationic lipid and cholesterol was developed for the delivery of a small-molecule chemotherapeutic drug, doxorubicin (Dox) to treat MDR tumor. The liposome-modified by ApoA1 was found to promote drug uptake and elicit better therapeutic effects than free Dox and liposome in MCF-7/ADR cells. Further, loading Dox into the present ApoA1-liposome systems enabled a burst release at the tumor location, resulting in enhanced anti-tumor effects and reduced off-target effects. More importantly, ApoA1-lip/Dox caused fewer adverse effects on cardiac function and other organs in 4T1 subcutaneous xenograft models. These features indicate that the designed liposomes represent a promising strategy for the reversal of MDR in cancer treatment.

## 1. Introduction

Chemotherapy is one of the main therapeutic approaches for cancer treatment and includes the application of small-molecule drugs, such as anthracyclines, topoisomerase inhibitors and antimetabolites. The occurrence of multidrug resistance (MDR) is a major reason for the failure of chemotherapy [1]. The main cause of MDR is the increased efflux of anti-cancer drugs by membrane-embedded drug transporters [2]. The most common drug transporters including P-glycoprotein (P-gp), multidrug resistance protein 1 (MPR1), and breast cancer resistance protein (BCRP) belong to the ATP-Binding Cassette (ABC) superfamily, which depend on the consumption of adenosine triphosphate (ATP) [3,4].

Several strategies have been developed to tackle the MDR phenomenon. MDR transport inhibitors such as verapamil and cyclosporine A are applied to overcome MDR by binding and blocking the transport function of the transporter. The inhibition of P-gp can enhance the intracellular accumulation of anticancer drugs which are usually substrates of P-gp, including anthracyclines and vinca alkaloids [5,6]. Alternatively, to solve these problems, biochemical and physical methods as well as nanotechnologies are used to overcome MDR [2,7], such as microRNA and RNA interference, monoclonal antibodies targeting P-gp, polymeric nanoparticles, micelles, and liposome nanoparticles. Liposome nanoparticles are the most developed and widely applied technique, the applications of which can be observed in the FDA-approved nanodrugs Doxil^®^ and DaunoXome^®^ [8]. Doxil has been demonstrated to be able to exhibit antitumor activity on doxorubicin-resistant (MDR) C26 cells [9] and overcome drug resistance in a genetically engineered mouse model of breast cancer [10]. However, the cross-resistant between Dox and Doxil makes it ineffective in some tumors, such as sarcoma [11,12] and doxorubicin-resistant mouse B-cell leukemia [10], furthermore, a strong immune response and hypersensitivity syndrome [13] limit its clinical application.

Recently, reconstituted high density lipoprotein (rHDL) has been paid increasing attention as a drug carrier to improve treatment [14,15,16]. In vivo, HDL plays a crucial role in promoting cholesterol efflux from tissues to the liver for excretion through SR-B1 (scavenger receptor, class B type 1)-mediated endocytosis. It has been reported that SR-B1 is also highly expressed in many tumors, including breast cancer, colorectal cancer, and liver cancer, for the sake of getting more cholesterol to support rapid proliferation [17]. Thus, based on the proof of targeting SR-B1, HDL particles may effectively aggregate to the tumor site, and enhance the cell uptake of drugs, leading to an increased anti-tumor effect and reverse drug resistance. In addition, HDL particles have high security and are completely biodegradable and less immunogenic than PEGylated liposome, which has been reported to cause strong immune responses [18]. Moreover, HDL can escape from elimination by the reticuloendothelial system, and has a longer half-life period [17]. Due to the advantages of rHDL, in this study, we attempted to prepare the similar nanoparticles which were useful and safe for targeting drugs.

The surface charge is regarded as one of the most important properties of the nanoparticles. A positive surface charge is helpful for negatively charged cells to phagocytize the liposomes [19]. Functionalized cationic liposomes have been used to improve the antitumor efficacy and reversion of multidrug resistance [20]. Our laboratory has previously reported the preparation of rHDL with doxorubicin [21]. In this study, we prepared ApoA1-modified cationic liposomes loaded with doxorubicin and investigated the antitumor efficacy and reversion of multidrug resistance in breast cancer. Our data showed that liposomes modified by ApoA1 can enhance the drug intake and antitumor activity of liposomes both in vitro and in vivo. The results may be due to the combination of ApoA1 and SR-B1 and the positive surface charge of the liposomes promoting the drug influx and inhibiting drug efflux. Moreover, ApoA1 could realize the targeted delivery of drugs to MCF-7/ADR cells overexpressing SR-B1, and exhibited an antitumor effect by drug accumulation. This might provide a novel strategy for overcoming drug resistance in tumor therapy.

## 2. Results and Discussion

### 2.1. Preparation and Characterization of Liposomes

**Figure 1:**
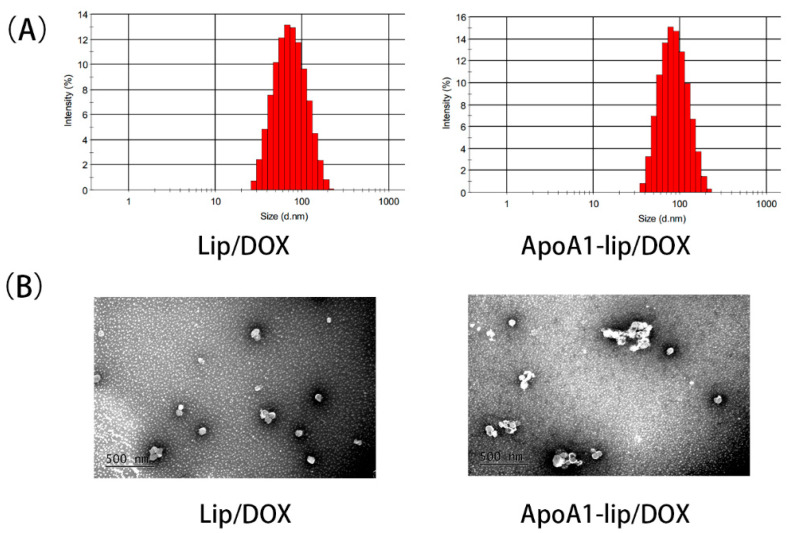
Size distribution (**A**) and observation by transmission electron microscopy (**B**) of Lip/Dox and ApoA1-lip/Dox.

According to previous studies, positively charged carriers could enhance the cellular drug accumulation [22]. ApoA1-lip/Dox and Lip/Dox liposomes were prepared with some modifications by incorporating the cationic phospholipid, (2,3-Dioleoyloxy-propyl)-trimethylammonium-chloride (DOTAP), into the liposomal formulation. As shown in Figure 1A,B, ApoA1-lip/Dox and Lip/Dox exhibited spherical morphology with a particle size of 80.6 ± 1.96 and 77.22 ± 1.35 nm and a uniform size distribution with a polydispersity index (PDI) of 0.156 ± 0.03 and 0.148 ± 0.02, respectively. The ξ-potential of ApoA1-lip/Dox was 6.23 ± 0.56 mV, which was lower than that of Lip/Dox at 13.56 ± 1.23 mV because of the negative charge of ApoA1. The encapsulation efficiencies of ApoA1-lip/Dox and Lip/Dox were 95.8 ± 0.63% and 96.5 ± 0.47%, respectively.

**Figure 2:**
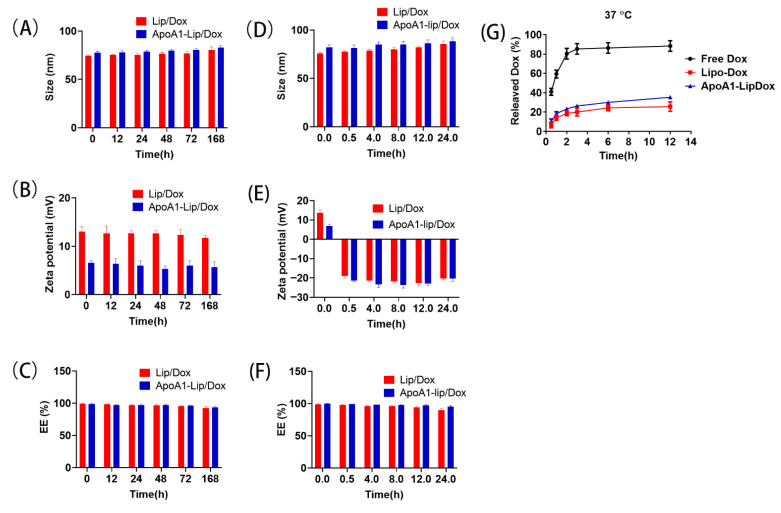
Stability of Lip/Dox and ApoA1-lip/Dox. Size distribution (**A**), zeta potential (**B**), and encapsulation efficiency (**C**) of Lip/Dox and ApoA1-lip/Dox stored at 4 ℃ for 168 h. Size distribution (**D**), zeta potential (**E**), and encapsulation efficiency (**F**) of Lip/Dox and ApoA1-lip/Dox cultivated in serum at 37 ℃ for 24 h. (**G**) The release profiles of Dox from free Dox, Lip/Dox, and ApoA1-lip/Dox at pH 7.4 PBS.

The liposomes were stored at 4 °C for a week. Figure 2A–C showed that no significant difference was found regarding the size and zeta potential of ApoA1-lip/Dox and Lip/Dox. The encapsulation efficiency (EE) remained at more than 90% in both liposomes. The size, zeta potential and EE also showed no significant difference when both liposomes were cocultured with plasma for 24 h (Figure 2D–F). The change of the zeta potential may have been due to the binding of cationic liposomes to anionic plasma proteins.

The release profiles of Dox from the drug-loaded liposomes were determined. The encapsulation of Dox by liposomes resulted in the sustained release of Dox. The release of Dox was about 25% from either ApoA1-lip/Dox or Lip/Dox at 37 °C within 12 h, while that of Dox was about 30% from ApoA1-lip/Dox (Figure 2G). By comparison, approximately 90% of Dox was released from all the free Dox within 12 h.

### 2.2. Cellular Uptake

**Figure 3:**
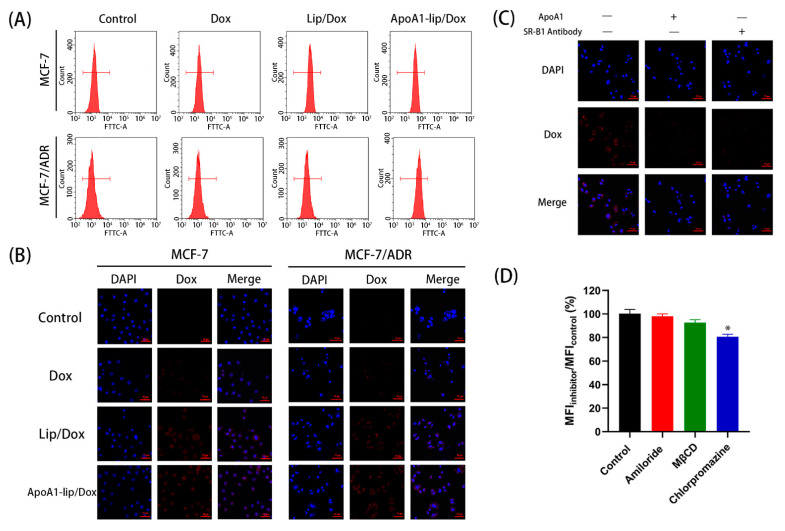
Cellular uptake profile of Dox-loaded liposomes. (**A**,**B**): Intracellular fluorescence of Dox measured by flow cytometry and a confocal laser scanning microscope (CLSM). The cells were co-incubated with different Dox liposomal formulations for 2 h. (**C**) Confocal observation after treating cells with free Dox, Lip/Dox, ApoA1-lip/Dox after pretreatment of 0.5 mg/mL anti-SR-B1 (scavenger receptor, class B type 1) antibody and ApoA1 for 2 h. (**D**) Effect of endocytosis inhibitors on cellular uptake. The percentage of cellular uptake was calculated by the Median Fluorescence Intensity (MFI) value of the inhibitor group normalized with that of the control group (100%). Cells without inhibitors were regarded as a control (*n* = 3; * *p* < 0.05, compared with control).

The cellular uptake of different Dox formulations was monitored in SR-B1-overexpressing MCF-7/ADR cells [23] (Figure S1) using Fluorescence-activated Cell Sorting (FACS) and confocal laser scanning microscope (CLSM), respectively. FACS results demonstrated that ApoA1-lip/Dox had the highest fluorescent intensity, while Lip/Dox showed higher fluorescent intensity than that of free Dox (Figure 3A). Figure 3B shows that CLSM showed similar results, in that the red fluorescence of the ApoA1-lip/Dox group could be clearly observed, and the Lip/Dox group showed a lower level of fluorescence, while the level of fluorescence in free Dox group was the lowest. The results indicated that the SR-B1-mediated endocytosis based on the active targeting effect of ApoA1 played an important role in the enhanced uptake of liposome compared to the electronic adsorptive endocytosis.

To further demonstrate the interaction between SR-B1 and ApoA1, we compared the cellular uptake of ApoA1-lip/Dox with or without SR-B1 antibody or free ApoA1 on MCF-7/ADR cells. The cells were pre-treated with SR-B1 antibody or excessive free ApoA1 to block the SR-B1 receptors on the cell membrane. As shown in Figure 3C, the cellular uptake of Dox in the presence of SR-B1 antibody or ApoA1 showed a significant decrease in MCF-7/ADR cells compared to the case without treatment with SR-B1 antibody or ApoA1. These results confirmed that ApoA1-lip/Dox was taken up by the cancer cells via the SR-B1-mediated internalization [17].

Endocytosis is one of the main ways for cells to take in liposomes, thus, different inhibitors were employed to elucidate the endocytosis pathways of ApoA1-lip/Dox (Figure 3D). Chlorpromazine (clathrin-mediated endocytosis inhibitor) [24] significantly decreased the cellular uptake of ApoA1-lip/Dox, showing that ApoA1-lip/Dox was internalized by the cells through the clathrin-mediated endocytosis pathway. Methyl-β-cyclodextrin (MβCD, caveolae-mediated endocytosis inhibitor) [24] showed a slight decrease in the cellular uptake of ApoA1-lip/Dox, while amiloride (macropinocytosis inhibitor) [24] did not have any inhibitory effect. The results suggested that another main endocytosis pathways of ApoA1-lip/Dox was clathrin-mediated endocytosis.

Taken together, liposomes modified with ApoA1 could be taken up into cells effectively through SR-B1-mediated endocytosis and clathrin-mediated endocytosis.

### 2.3. Cell Apoptosis and Cytotoxicity

**Figure 4:**
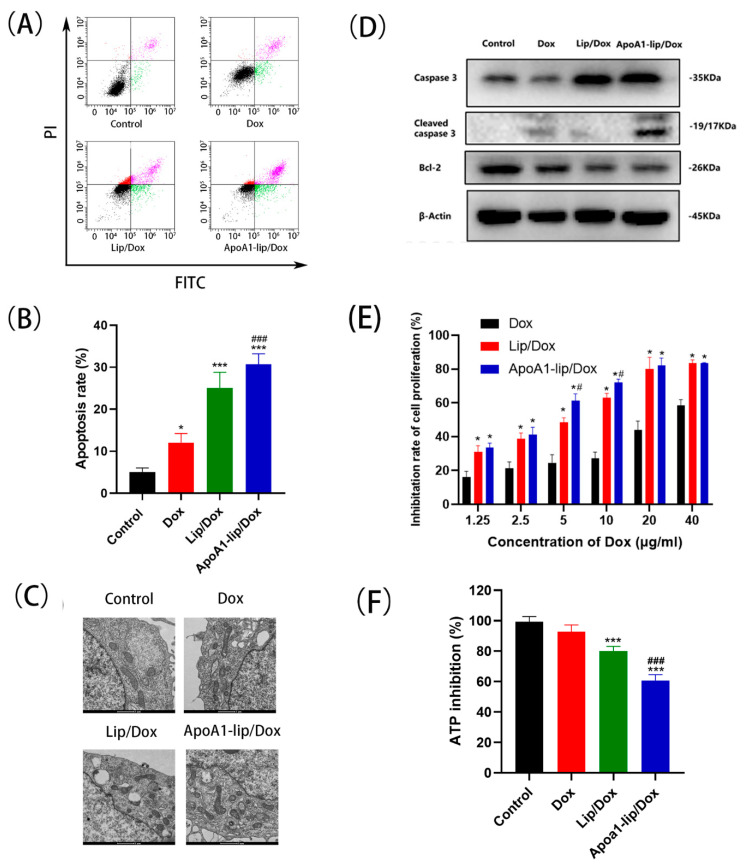
(**A**,**B**) Apoptosis of MCF-7/ADR cells induced by different Dox formulations after 12 h of incubation determined using the Annexin V-FITC/PI staining. (**C**) Transmission electron microscopical images of MCF-7/ADR cells treated with different drug formulations. (**D**) The levels of apoptotic related proteins in MCF-7/ADR after treatment with different formulations. (**E**) The inhibition rate of different Dox formulations on MCF-7/ADR cell proliferation. Data in the graph are presented as mean ± SD (*n* = 3). * *p* < 0.05 versus Dox group; # *p* < 0.05 versus Lip/Dox group. (**F**) The adenosine triphosphate (ATP) inhibition of different Dox formulations on MCF-7/ADR cells. *** *p* < 0.01 versus control group; ### *p* < 0.01 versus Dox group.

Apoptosis is one of the major modes of cell death in response to chemotherapy. The apoptosis-inducing effect of ApoA1-lip/Dox was evaluated using annexin V-FITC/PI apoptosis detection kit [25]. As shown in Figure 4A,B, ApoA1-lip/Dox had the most effective apoptosis-inducing capacity compared to other drug formulations. Free Dox induced 12.1% of cell apoptosis and Lip/Dox, and ApoA1-lip/Dox induced 25.2% and 30.7% of MCF-7/ADR cell apoptosis, respectively, leading to a 2.08- and 2.53-fold increase compared with free Dox treated cells. The results explained that the liposomes could increase cell apoptosis by enhancing intracellular uptake and further resulted in better antitumor efficacy.

Further, we investigated whether apoptosis induced by liposomes was mediated by the mitochondrial pathway. The mitochondrial is a key pathway related to apoptosis [26]. As shown in Figure 4C, the apoptosis-inducing effect of Lip/Dox was more efficient than that of Dox, and the most severe mitochondrial ultrastructural injury was observed in the ApoA1-lip/Dox.

Moreover, several apoptosis-related proteins were detected to further reveal the effect of cell apoptosis. As shown in Figure 4D and Figure S3, compared with the control group, levels of Bcl-2, an anti-apoptotic protein, were significantly downregulated in the Lip/Dox and ApoA1-lip/Dox groups. Furthermore, the levels of cleaved caspase-3 and caspase-3 were upregulated when treated with ApoA1-lip/Dox, exceeding the levels of the free doxorubicin group.

The cytotoxicity of ApoA1-lip/Dox was calculated against MCF-7/ADR and MCF-7 cells by an MTT assay. As shown in Figure 4E and Table S1, compared with Dox group, Lip/Dox and ApoA1-lip/Dox showed slightly increased cytotoxicity on MCF-7 cells, whereas ApoA1-lip/Dox showed significantly higher cytotoxicity toward MCF-7/ADR cells after 96 h of incubation. Meanwhile, the half maximal inhibitory concentration (IC50) of Lip/Dox was 7.87 μg/mL, leading to a 3.67-fold reduction compared to Dox. The results demonstrated that Dox encapsulated in liposomes and the positive potential enhanced the cytotoxicity of Dox and partly reversed MDR, which was mainly due to bypassing the efflux of P-gp through endocytosis [22]. In addition, the IC50 of ApoA1-lip/Dox was 4.38 μg/mL, which showed significantly greater cytotoxicity than that of Lip/Dox, indicating that MDR could be further overcome by an ApoA1-liposome delivery system for chemotherapeutic agents. Moreover, the resistant index (RI) values of Lip/Dox and ApoA1-lip/Dox were 13.57 and 9.13, showing a 3.01- and 4.47-fold decrease compared with Dox (40.78), respectively, and the reversal factor (RF) values of Lip/Dox and ApoA1-lip/Dox were 3.73 and 6.70, respectively. These results further suggest that wrapping Dox in liposomes can significantly improve the sensitivity of drug-resistant cells and overcome MDR to some extent.

Again, ATP inhibition was also detected by an ATP assay kit. ATP, as the most important energy molecule, plays an important role in various physiological and pathological processes of cells. Normally, the ATP level will decrease when the cell is in apoptosis or necrosis, which also indicates impaired or decreased mitochondrial function [27]. ApoA1-lip/Dox exhibited distinctively marked ATP inhibition, followed by Lip/Dox treatments and free Dox showed low inhibition in ATP production after 48 cultivation, indicating that the liposomes systems exert stronger inhibitory effects on the cancer cells (Figure 4F).

Accordingly, for the effect of ApoA1-lip/Dox on MCF-7/ADR, the targeting effect of SR-B1 and the positive charge of cationic liposomes significantly promoted cellular uptake, the accumulation of Dox induced cell apoptotic, impaired mitochondrial function, reduced ATP level, thus inhibiting the transport of P-gp, and resulted in significant enhancement of Dox on cytotoxicity and reversal of resistance.

### 2.4. In Vivo Biodistribution

**Figure 5:**
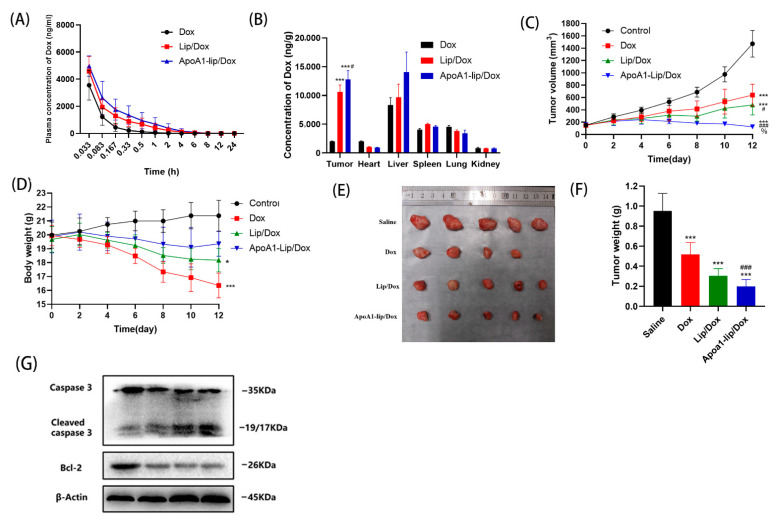
(**A**) Plasma concentration-time curves of Dox in rats after intravenous administration with different Dox formulations at a dose of 5 mg/kg Dox (*n* = 4, mean ± SD). (**B**) In vivo biodistribution of Dox in 4T1 tumor-bearing mice at 2 h after intravenous administration with Dox, Lip/Dox, and ApoA1-lip/Dox. Data in the graph are presented as mean ± SD (*n* = 4). *** *p*< 0.01 versus Dox group; # *p* < 0.05 versus Lip/Dox group. (**C**,**D**) The tumor volume and body weight of tumor bearing mice were measured every two days after being treated with different formulations at a dose of 5 mg Dox/kg. * *p* < 0.05, *** *p* < 0.01 compared with control, ^#^ *p* < 0.05, ^###^ *p*< 0.01, compared with Dox group, % *p* < 0.05 compared with Lip/Dox group. (**E**,**F**) A representative photograph and average weight of removed tumor from the sacrificed mice at the end of the experiment. (**G**) The levels of apoptotic related proteins in tumor after treatment with different formulations.

The pharmacokinetics of each Dox formulation were investigated after intravenous administration in rats. The plasma concentration-time curves and the pharmacokinetic parameters are presented in Figure 5A and Table S2, respectively. Following single dose administration, ApoA1-lip/Dox displayed the slowest decay in the plasma concentration of drugs compared to free Dox and Lip/Dox, substantiated by the higher area under the curve (AUC), longer t_1/2_ and mean residence time (MRT), and lower Cl, which allowed improved tumor targetability and therapeutic index in vivo.

Several studies showed the good tumor targeting properties of rHDL [15,28]. The biodistribution of administered Dox formulations was also explored in major organs including the heart, liver, spleen, lung, kidney, and tumor using an SR-B1- overexpressing 4T1 tumor bearing model (Figure S2). As shown in Figure 5B, the drug accumulation in the tumor sites of mice treated with Lip/Dox or ApoA1-lip/Dox was much higher than that of the mice injected with free Dox. The outcome was very likely due to (1) the long blood circulation of Lip/Dox or ApoA1-lip/Dox and the permeability and retention (EPR) effect result in passively targeted delivery to tumor sites; (2) the surface modification of ApoA1 which helped the drugs to better escape the “reduced reticuloendothelial system” (RES) organs [29,30].

### 2.5. Antitumor Effects In Vivo

As mentioned above, liposomes modified with ApoA1 could enhance the cell uptake of Dox and reduce toxicity, and were able to increase the delivery to tumor sites, the key question was therefore whether these advantages could be transferred to effective in vivo anticancer treatments. Therefore, the antitumor efficacy of different Dox formulations was detected in in mice bearing 4T1 tumor.

As shown in Figure 5C, the tumor volume in mice treated with saline grew rapidly to more than 1000 mm^3^ on the 12th day. Moreover, the tumor growth curve showed that ApoA1-lip/Dox had the most significant inhibitory effect on tumor volume, which was mainly attributed to the enhanced permeability and retention effect (EPR) and tumor-targeting capability of the liposomes functionalized by ApoA1, and Lip/Dox and free Dox repressed tumor growth as well. At the end of the experiment, the tumors were harvested, weighed, and photographed. As shown in Figure 5F, ApoA1-lip/Dox showed the most significant inhibitory effect in the treatment groups (*p* < 0.01 compared with free Dox and *p* < 0.05 compared with Lip/Dox), which was consistent with the results of the tumor volume and the tumor inhibition rates were 45.6%, 68.1, and 79.2% for free Dox, Lip/Dox and ApoA1-lip/Dox, respectively.

**Figure 6:**
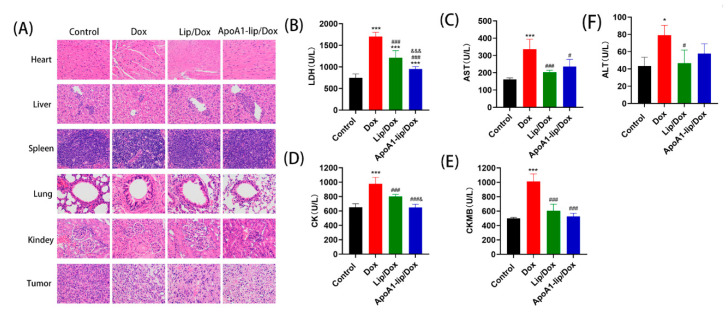
(**A**) H&E staining of major organs and tumor sections harvested from tumor-bearing mice on the 12th day after treated with saline, Dox, Lip/Dox, and ApoA1-lip/Dox. (**B**–**F**) The levels of lactate dehydrogenase (LDH), aspartate aminotransferase (AST), creatine kinase (CK), creatine kinase/MB isoenzyme (CK-MB) and alanine aminotransferase (ALT) after intravenous administration with different Dox formulations in 4T1-bearing mice. (*n* = 5, mean ± SD, * *p* < 0.05, *** *p* < 0.01, compared with saline group, ^#^ *p* < 0.05, ^###^ *p* < 0.01, compared with Dox, ^&^ *p* < 0.05, ^&&&^ *p* < 0.01, compared with Lip/Dox.

H&E staining was used to further analyze the effect of drug formulations on tumors. As shown in Figure 6A, in the saline group, tumors were composed of tightly packed tumor cells interspersed with various amounts of stroma. However, Lip/Dox and ApoA1-lip/Dox group triggered extensive tumor necrosis, while free Dox could also induce tumor necrosis slightly compared with the control.

Further, the expression of apoptosis-related proteins was also examined in tumor tissues. As shown in Figure 5G and Figure S3, when treated with Lip/Dox and ApoA1-lip/Dox, the levels of Bcl-2 were obviously down-regulated and the levels of cleaved caspase-3 were significantly up-regulated compared with the control group. These results agree with the results in vitro.

### 2.6. Safety Evaluation

The severe systemic toxicity of Dox has always been a major obstacle to its clinical application [31], therefore, the body weight was measured every other day to assess the systemic side effects of different Dox preparations. Figure 5D shows that the body weight of mice treated with saline increased slightly due to the rapid tumor growth. However, a significant weight loss was observed during the free Dox treatment and one mouse died before the end of the experiment, which indicated the severe systemic toxic effects of Dox. Moreover, a slightly reduced animal weight was observed in the Lip/Dox group at the end of the treatment, but no remarkable change in body weight was observed during the treatment with ApoA1-lip/Dox, demonstrating that the drugs encapsulated in liposomes were relatively safer than using free Dox.

To further assess the system toxicity of the Dox formulations, we harvested the primary organs from mice treated with the drugs and analyzed them by histological staining.

Cardiotoxicity is one of the most serious side effects of Dox. As shown in Figure 6A, for the Dox treatment group, the heart showed obvious pathological damage such as membranolysis, nucleus necrosis and cardiac fibrosis, and alveolar injury, minimal hemorrhage, and cell apoptosis were also observed in other tissues, demonstrating the serious system toxicity of Dox. However, H&E results show that Lip/Dox and ApoA1-lip/Dox treated-mice had no obvious damage in these tissues compared with the control group. These results further prove the safety of ApoA1-lip/Dox.

Lactate dehydrogenase (LDH), aspartate aminotransferase (AST), creatine kinase (CK), and creatine kinase/MB isoenzyme (CK-MB), as markers of cardiac toxicity, indicate the level of cardiac function. AST and alanine aminotransferase (ALT) can reflect the level of liver function. As shown in Figure 6B–F, compared with the saline group, the levels of LDH, AST, CK, CK-MB and ALT exhibited significant difference in Dox groups, which was consistent with the results of H&E staining indicated previously. The activities of AST, CK, CK-MB and ALT were remarkably lower in the serum of Lip/Dox and ApoA1-lip/Dox treated-mice as compared with free Dox treated mice, and were close to those of saline group. The values of LDH were somewhat higher in the serum of the ApoA1-lip/Dox group than that of the control group, but were lower than those of free Dox and Lip/Dox groups. These findings agreed with the results from an in vivo drug distribution study that showed that free Dox accumulated to a great extent in the heart, while ApoA1-lip/Dox had an effective targeting effect on tumor. (Figure 5B). All of these data suggest that liposomes modified with ApoA1 are safe and effective delivery carriers for tumor chemotherapy drugs.

**Figure 7:**
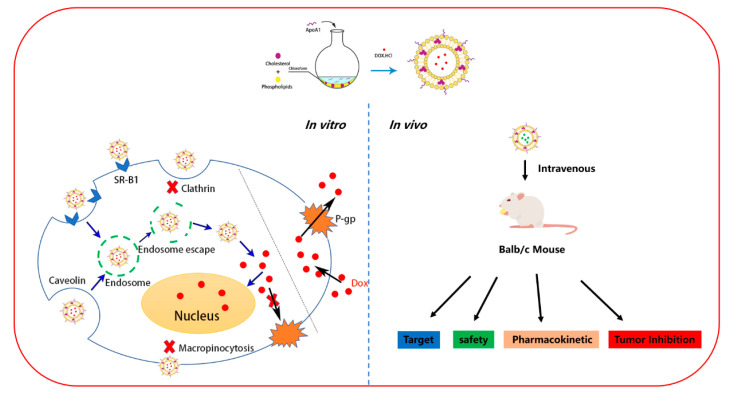
Schematic illustration of the preparation, the antitumor mechanism, and safety evaluation of ApoA1-lip/Dox.

Although chemotherapy is one of the most effective methods of tumor therapy, tumor cell acquisition of drug resistance is one of the major obstacles for efficacious chemotherapy. In this study, we evaluated the antitumor effects and mechanism of action of rHDL-based nanoparticle (ApoA1-lip/Dox) against drug-resistant breast cancer cells. rHDL has been studied for many years with good stability, low cytotoxicity, and high targeting efficiency [17,32]. Here, ApoA1-lip/Dox was discrete and spherical in shape and displayed a good size distribution. Results in Figure 3 and Figure 4 showed that ApoA1-lip/Dox could effectively overcome the drug efflux effect of P-gp and accumulate more drugs in MCF-7/ADR cells through SRB1-mediated endocytosis and clathrin-mediated endocytosis which induced more apoptosis and cytotoxicity (Figure 7). The results in Figure 5 and Figure 6 show that ApoA1-lip/Dox possessed a favorable tumor-targeting quality, promising antitumor activity, and reasonable safety which was similar to those of other studies [32,33,34].

## 3. Materials and Methods

### 3.1. Materials

Doxorubicin hydrochloride (Dox·HCl) was purchased from Dalian Meilun Biotechnology Co. Ltd. (Liaoning, China). (2,3-Dioleoyloxy-propyl)-trimethylammonium-chloride (DOTAP), egg yolk lecithin (EPC) and cholesterol (Chol) were purchased from A.V.T. Pharmaceutical Tech Co., Ltd. (Shanghai, China). Apolipoprotein A1(ApoA1) was acquired from RAAS Blood Products Co., Ltd. (Shanghai, China). Roswell Park Memorial Institute (RPMI) 1640 medium was bought from Dalian Meilun Biotechnology Co. Ltd. (Liaoning, China). Fetal bovine serum was purchased from Gibco (Carlsbad, CA, USA). 4,6-diamidino-2-phenylindole (DAPI) were from Sigma (St. Louis, MI, USA). All the primary antibodies were bought from Abcam (Cambridge, MA, USA).

### 3.2. Preparation and Characterization of Liposome

Lip/dox and ApoA1-lip/Dox were prepared using the thin-film method [35]. Briefly, DOTAP, EPC and Chol were weighed at a ratio of DOTAP:EPC:Chol = 25:20:18 (*w*:*w*) and dissolved in the mixture of chloroform:methanol (2:1, *v*:*v*), followed by the evaporation of the mixture under a vacuum at 40 °C to form a thin film layer. The film was totally evaporated under vacuum overnight and rehydrated with 250 mM ammonia sulfate solution with a rotary evaporator at 40 °C at low speed. Then the mixture was ultrasonicated for 20 min under an ice bath at 200 W and filtered through a 0.22 μm microfiltration membrane to remove the debris and particles with large sizes. The ammonium sulfate gradient was established by loading the solution onto the Sephadex G-50 gel column (Shanghai yuanye Bio-Technology Co., Ltd, Shanghai, China). ApoA1-liposome was obtained by adding ApoA1 at the molar ratio of lipid to ApoA1 of 60:1 at 37 °C for 1 h. Lip/dox and ApoA1-lip/Dox were established by incubating Dox with liposomes and ApoA1-liposomes (Shanghai RAAS, Shanghai, China) at 55 °C for 1 h. Free Dox was removed by dialysis (MWCO 8–14 kDa) (Shanghai yuanye Bio-Technology Co., Ltd, Shanghai, China). Lip/Dox and ApoA1-lip/Dox were lysed with 1% Tritonx-100 (Meilun Bio-technology Co., Ltd, Dalian, China) at 37 °C for 0.5 h. Then the amount of Dox was evaluated by Tecan M1000 Multi-Mode Microplate Reader (TECAN, Männedorf, Switzerland) at an Ex of 488 nm and Em of 590 nm. The average particle size, polydispersity index (PDI) and zeta potential of the liposomes were analyzed by a Zetasizer Nano (Malvern Instruments, Malvern, Worcestershire, UK).The encapsulation efficiency (EE) was calculated according to the following equation: EE (%) = W_Encapsulated-drug_/W_Total-drug_ × 100%.

The morphology of liposomes was examined via a JEM 1200 transmission electron microscope (JEOL JEM-1200EX, JEOL, Tokyo, Japan) using 2% uranyl acetate for negative staining.

### 3.3. Stability of Liposome

The storage stability of the drug-loaded liposomes was determined by detecting the changes in particle size, zeta potential and drug leakage for one week at 4 ℃.

The plasma stability of the drug-loaded liposomes was also determined by monitoring the variation in particle size, zeta potential and drug leakage. Briefly, the liposomes were mixed with the plasma (1:1, *v*:*v*) and incubated at 37 ℃, the samples were collected at different incubation times (0, 4, 8, 12 and 24 h) and then free Dox was removed by dialysis (MWCO 8–14 kDa). Lip/Dox and ApoA1-lip/Dox were pyrolyzed with 1% Triton-100 at 37 ℃ for 0.5 h. Then, Dox was excited at 488 nm and emitted at 590 nm using Tecan M1000 Multi-Mode Microplate Reader.2.4.

### 3.4. In Vitro Drug Release

The in vitro release of Dox from liposomes was investigated by the dialysis bag diffusion technique. Briefly, 1 mL of liposome solution was placed in dialysis bags (MWCO 8−14 kDa) and immersed into 40 mL of PBS (pH 7.4). The entire system was performed at 37 °C under continuous shaking at 100 rpm. At a predetermined time interval, 1 mL of samples was withdrawn and replaced with fresh PBS. The drug concentrations were detected as described above.

### 3.5. Cell Culture and Animals

Human breast adenocarcinoma (MCF-7) cells, and multidrug resistant MCF-7 (MCF-7/ADR) cells [36] were kindly provided by the Department of Pharmacy, at the School of Pharmacy, Fudan University. MCF-7 cells and MCF-7/ADR cells were cultured in RPMI-1640 medium with 10% FBS, 100 U/mL of penicillin, and 100 μg/mL of streptomycin at 37 °C with 5% CO_2_. The drug resistance of MCF-7/ADR cells was maintained by the addition of Dox (1 μg/mL) in the medium. The 4T1 cells were cultured in RPMI-1640 with 10% FBS, 1% penicillin and streptomycin and incubated under standard sterile conditions for cell cultures (5% CO_2_, 37 °C). The cell passage was as follows: when the cells were 80–90% confluent, the old solution was discarded, they were washed with PBS and 2 mL of digestion solution (0.25% trypsin + 0.03% EDTA) was added for digestion. It was observed under the microscope that the cells were completely separated from the bottle wall and separated into single cells, then the trypsin was discarded, and the medium added and mixed evenly. The culture medium was added to 6–8 mL in T25 bottle, and cultured in a 37 °C, 5% CO_2_ incubator. All cell culture experiments were done in 3 independent repeats with cells passaged less than 3 times.

Animals were provided by the Shanghai Slack Laboratory Animal Co., Ltd. (Shanghai, China). The animal care and animal experimentations were performed according to the Guide for the Care and Use of Laboratory Animals. The experiments were carried out in accordance with the guidelines issued by the Ethical Committee of Fudan University with permission number 2019000521893.

### 3.6. Flow Cytometry Study

MCF-7/ADR cells were seeded in six-well plates at the density of 5 × 10^5^/mL and cultured for 24 h. After moving the culture media, the cells were added to different formation of Dox for 4 h. The cells were washed with PBS three times and were analyzed using flow cytometer (Beckman, Hightstown, NJ, USA) excited at 488/590 nm.

### 3.7. Confocal Microscopy Observation

The cells were cultured in 35 mm culture dishes (Nest, Wuxi, China) for 24 h. Different formulations (Dox concentration 5 mg/mL) were incubated with the cells for 2 h. Then, 4′,6-diamidino-2-phenylindole (DAPI) probes were added for 10 min. The cells were washed with PBS three times and observed using a confocal laser scanning microscope (CLSM, Zeiss, Olympus, LSM710, Carl Zeiss Jena, Germany). Dox was excited at 488 nm and emitted at 590 nm and DAPI was excited at 340 nm and emitted at 488 nm.

### 3.8. Endocytosis Inhibition

The endocytosis pathway of the liposomes was detected. The cells were cultured in 24-well plates at the density of 10^5^/well for 24 h. Then the cells were pretreated with anti-SR-B1 antibody (ab217318, 0.5 μg/mL), and ApoA1 (0.5 μg/mL) for 1 h, and ApoA1-lip/Dox (Dox concentration 5 mg/mL) containing medium was added for 2 h at 37 °C. Cellular uptake was observed by confocal microscopy (Zeiss-LSM710; Carl Zeiss Jena, Germany). Then, the cells were pretreated with Chlorpromazine (30 μM), MβCD (5 mM), Amiloride (50 μM) for 1 h, and different liposome preparations containing medium were added for 2 h at 37 °C [24]. The endocytosis pathway of the liposomes was detected. The cells were cultured in 24-well plates at the density of 10^5^/well for 24 h. Then the cells were pretreated with anti-SR-B1 antibody (ab217318, 0.5 μg/mL), and ApoA1 (0.5 μg/mL) for 1 h, and ApoA1-lip/Dox (Dox concentration 5 mg/mL) containing medium was added for 2 h at 37 °C. Cellular uptake was observed by Confocal microscopy. Additionally, the cells were pretreated with sucrose (154 mg/mL), nystatin (15 μg/mL), and amiloride (133 μg/mL) for 1 h, and different liposome preparation containing media were added for 2 h at 37 °C. The cellular uptake was determined by intracellular fluorescence intensity detected by flow cytometry and the relative uptake efficiency was calculated by the comparison of intracellular fluorescence intensity. The data were analyzed using CytExpert 2.3 software (Beckman Coulter, Brea, CA, USA).

### 3.9. Cell Apoptosis

To evaluate the apoptosis-inducing capability of different liposomes, an Annexin V-FITC/PI apoptosis detection kit (Beyotime, Shanghai, China) was used to identify and quantitate apoptotic cells. The cells were cultured in six-well plates for 24 h and were then incubated with different liposomes for 24 h, then the cells were collected and 5 × 10^5^ cells were stained with annexin-V-FITC (Beyotime Biotechnology, Shanghai, China) for 10 min at room temperature. The cells were centrifuged at 1000× *g* for 5 min at room temperature. After being washed once with PBS, the cells were stained with propidium iodide (PI). The percentage of apoptotic cells was analyzed by CytoFLEX flow cytometer immediately. The data were analyzed using CytExpert 2.3 software. MCF-7/ADR cells were cultivated in the six-well plate and treated with different Dox formulations. Then, cells were collected and processed according to the method reported previously [37]. Briefly, cells were fixed with 2% glutaraldehyde in RPMI-640 medium for 15 min, and then fixed in 2% glutaraldehyde with 0.1 M Na cacodylate/HCl (pH7.4) for 30 min. After extensive washing in 0.2 M Na cacodylate/HCl (pH7.4) three times, cells were fixed with 1% OsO4–0.15 M Na cacodylate/HCl (pH7.4) for 30 min. The cells were then dehydrated in an increasing gradient of ethanol and polymerized at 60 °C for 48 h. A JEM 1200 transmission electron microscope (JEOL JEM-1200EX, JEOL, Tokyo, Japan) was used to analyze the samples at 60 kV.

### 3.10. In Vitro Cytotoxicity

The cytotoxicity of different liposomes to MCF-7 and MCF-7/ADR cells was examined using an MTT assay. The cells were seeded at a density of 5 × 10^3^ cells/well in 96-well plates and incubated overnight and were then co-incubated with different liposomes for 96 h. Afterwards, the cells were incubated with 0.5 mg/mL MTT for a further 4 h, and the supernatant was removed and DMSO was added to each well. The absorbance was measured at a wavelength of 570 nm using a microplate reader and a reference wavelength of 630 nm was followed. Cytotoxicity data were analyzed by GraphPad Prism 8 (GraphPad Software Inc, San Diego, CA, USA) to determine the 50% inhibitory concentration (IC50) in triplicate.

### 3.11. Inhibition of Intracellular ATP Production

ATP concentrations were determined by using an Enhanced ATP Assay Kit (Beyotime, Shanghai, China) according to the manufacturer’s protocol. Briefly, MCF-7/ADR cells were seeded at 4 × 10^5^ cells/well in six-well plates. After being incubated with ApoA1-lip/Dox (5 mg/mL) for 24 h, cells were rinsed and lysed using ATP lysis buffer on ice. Samples were collected and centrifuged at 12,000 rpm for 10 min at 4 °C to acquire the supernatant for further determination. Samples and ATP detection working dilution were added and luminescence activity was measured immediately using luminometer (Promega Corporation, Madison, WI, USA). A standard curve of ATP measurements was made in each assay. Subsequently, the intracellular ATP contents were normalized by the protein contents in each sample. The data and images were processed by GraphPad Prism 8 statistical software (GraphPad Software Inc, San Diego, CA, USA).

### 3.12. Western Blot Analysis

The cells were seeded into six well plates at a density of 5 × 10^5^/well and treated with different Dox formulations (Dox 5 μg/mL) for 48 h. Cells were lysed in RIPA buffer supplemented with PMSF on ice. Equal amounts of proteins (20 μg) were separated using 10% NuPAGE Bis-Tris Pre-Cast Gels and then transferred to 0.45 μm polyvinylidene fluoride (PVDF) membranes (PerkinElmer). The PVDF membranes were blocked with 5% nonfat milk for 1 h and incubated with primary antibodies overnight at 4 °C. HRP conjugated secondary antibodies were incubated for 2 h at room temperature. Finally, immunoreactive bands were visualized with an ECL Plus kit and imaged with a ChemiDoc Touch Imaging System (Bio-Rad Laboratories, Hercules, CA, USA). β-actin was used as an internal control. The semi-quantification of the immunoblotting was performed using ImageJ 1.52c (NIH, Bethesda, Maryland, MD, USA).

### 3.13. In Vivo Pharmacokinetics

The Sprague-Dawley rats were randomly divided into three groups (*n* = 4), and injected intravenously with Dox, Lip/Dox and ApoA1-lip/Dox at the dose of 5 mg/kg Dox. After administration, the blood samples were collected in a heparinized tube at 0.033, 0.083, 0.25, 0.5, 0.75, 1, 2, 4, 8,12, and 24 h and then separated by centrifugation at 4000 rpm for 10 min. Plasma samples were extracted by methanol to remove protein before being analyzed by the LC/MS system. Dox was eluted using a gradient of solvent (A) 0.1% formic acid in Milli-Q water and solvent (B) 0.1% formic acid in acetonitrile. The flow rate was 0.3 mL/min, and the injection volume was 20 μL. The reversed phase-high liquid chromatography (RP-HPLC) C18 column (Shimadzu, Tokyo, Japan, 50 mm× 4.6 mm, 3 mm particle size) was used in the analysis and ultraviolet detection was set at a wavelength of 488 nm.

The pharmacokinetic parameters were calculated by Phoenix winNonlin software (Pharsight Corporation, CA, USA), such as the area under the plasma concentration time curve (AUC_0-∞_), elimination half-life (t_1/2_), clearance (Cl) and mean residence time (MRT).

### 3.14. Biodistribution

When the tumors reached 100–200 mm^3^, 4T1 tumor-bearing balb/c mice received an intravenous injection (200 μL) of Dox, Lip/dox and ApoA1-lip/Dox (Dox 10 mg/kg, six mice each group). After 2 h administration, the mice were sacrificed, and the heart, liver, spleen, lung, kidney, and tumor were removed, weighed, and then extracted with methanol by the smashing of tissues and organs, followed by centrifugation at 12,000 rpm for 10 min and analysis by LC-MS/MS according to the above method.

### 3.15. In Vivo Antitumor Efficacy

The 4T1-bearing BALB/c mice were divided into five groups when the tumors grew to about 80–100 mm^3^, and were intravenously administrated with 200 μL of saline, Dox, Lip/Dox, and ApoA1-lip/Dox, the concentration of Dox was maintained at 1 mg/mL. The tumor size and body weight of mice were measured every other day during the treatment for 2 weeks.

### 3.16. Statistical Analysis

Results are presented as mean ± standard deviation (S.D.). A student t-test or one-way ANOVA was applied to test for significance in the experiments. Statistical significance was set at * *p* < 0.05 while extreme significance was set at *** *p* < 0.01.

## 4. Conclusions

In summary, our study demonstrated that ApoA1-lipsome, as a kind of biological drug-delivery carrier, is highly effective in transporting the anti-cancer drugs doxorubicin into MCF-7/ADR cells. In addition, the encapsulation of Dox in ApoA1-lipsome could significantly enhance cellular uptake via EPR effects and SB-B1 targeting effects, increase the cytotoxicity, induce cell apoptosis, and reverse MDR. Moreover, ApoA1-lip/Dox had a longer half-life and enhanced tumor targeting, which enhanced the anti-tumor effect of Dox with no obvious toxicity in vivo. Therefore, our study provides a feasible strategy to boost the therapeutic effect of breast cancer therapy by using the ApoA1-lipsome drug-delivery system.

## Data Availability

The data presented in this study are available from the corresponding author upon request.

## Supplementary Materials

The following are available online, Figure S1: Expression level of SR-B1 receptor in MCF7/ADR cells and MCF7 cells; Figure S2: Expression level of SR-B1 receptor in4T1 cells; Figure S3: Data of western blot; Table S1: IC50 values of DOX, Lip/Dox, ApoA1- Lip/Dox in MCF-7 and MCF-7/ADR cells after 96 h incubation and the resistant index (RI) and the reversal factor (RF) of different formulations at 96 h in MCF-7/ADR cells; Table S2: Pharmacokinetic parameters of Dox administered to animals as Dox, Lip/Dox and ApoA1-lip/Dox at a dose of Dox 5 mg/kg.

## Sample Availability

Not available.
