# Supplementary material for: Reversal of Multidrug Resistance by Apolipoprotein A1-Modified Doxorubicin Liposome for Breast Cancer Treatment
Source: Molecules. 2021 Feb 26;26(5):1280. doi: 10.3390/molecules26051280 (PMC7956628; doi:10.3390/molecules26051280)
Supplement: Supplementary file 1 [file molecules-26-01280-s001.zip › Supplementary materials/Figure S legends.docx]

Fig. S1 Expression level of SR-B1 receptor in MCF7/ADR cells and MCF7 cells. Jurkat cells and HepG2 cells were chosen as the negative and positive control, respectively.

Fig. S2 Expression level of SR-B1 receptor in4T1 cells. Jurkat cells and HepG2 cells were chosen as the negative and positive control, respectively.

Fig. S3 A, B and C: Data of western blot from Figure 4E in the graph are presented as mean ± SD (n = 3). *p < 0.05, ***p< 0.01 versus control; #p < 0.05, ###p < 0.05 versus Dox group; %p p < 0.05 versus Lip/Dox group. D, E and F: Data of western blot from Figure 5G in the graph are presented as mean ± SD (n = 3). *p < 0.05, ***p< 0.01 versus control; #p < 0.05, ###p < 0.01 versus Dox group.
