# Supplementary material for: Reversal of Multidrug Resistance by Apolipoprotein A1-Modified Doxorubicin Liposome for Breast Cancer Treatment
Source: Molecules. 2021 Feb 26;26(5):1280. doi: 10.3390/molecules26051280 (PMC7956628; doi:10.3390/molecules26051280)
Supplement: Supplementary file 1 [file molecules-26-01280-s001.zip › Supplementary materials/Table S1.docx]

| Formulations | IC50 (lg/mL) | | RI | RF |
| --- | --- | --- | --- | --- |
|  | MCF7 | MCF7/ADR |  |  |
| DOX | 0.72 | 29.36 | 40.78 |  |
| Lip/Dox | 0.58 | 7.87 | 13.57 | 3.73 |
| ApoA1- Lip/Dox | 0.48 | 4.38 | 9.13 | 6.70 |

Table S1 IC50 values of DOX, Lip/Dox, ApoA1- Lip/Dox in MCF-7 and MCF-7/ADR cells after 96 h incubation and the resistant index (RI) and the reversal factor (RF) of different formulations at 96 h in MCF-7/ADR cells.
