# Supplementary material for: Reversal of Multidrug Resistance by Apolipoprotein A1-Modified Doxorubicin Liposome for Breast Cancer Treatment
Source: Molecules. 2021 Feb 26;26(5):1280. doi: 10.3390/molecules26051280 (PMC7956628; doi:10.3390/molecules26051280)
Supplement: Supplementary file 1 [file molecules-26-01280-s001.zip › Supplementary materials/Table S2.docx]

| Parameter |  | DOX | Lip/Dox | ApoA1-lip/Dox |
| --- | --- | --- | --- | --- |
| AUC(0-t) | h·mg/L | 551.5317 | 1946.707 | 2887.578 |
| AUC(0-∞) | h·mg/L | 551.9693 | 1959.083 | 2888.921 |
| MRT(0-t) | h | 1.346 | 4.807 | 6.471 |
| MRT(0-∞) | h | 1.865 | 6.073 | 8.498 |
| t_1/2_ | h | 2.275 | 4.656 | 6.127 |
| t_max_ | h | 0.033 | 0.033 | 0.033 |
| CL | L/h/kg | 0.0091 | 0.0026 | 0.0017 |
| C_max_ | mg/L | 3.554667 | 4.659333 | 5.309333 |

Table S2 Pharmacokinetic parameters of Dox administered to animals as Dox, Lip/Dox and ApoA1-lip/Dox at a dose of Dox 5 mg/kg.
