# Supplementary figures and images for: Reversal of Multidrug Resistance by Apolipoprotein A1-Modified Doxorubicin Liposome for Breast Cancer Treatment
Source: Molecules. 2021 Feb 26;26(5):1280. doi: 10.3390/molecules26051280 (PMC7956628; doi:10.3390/molecules26051280)

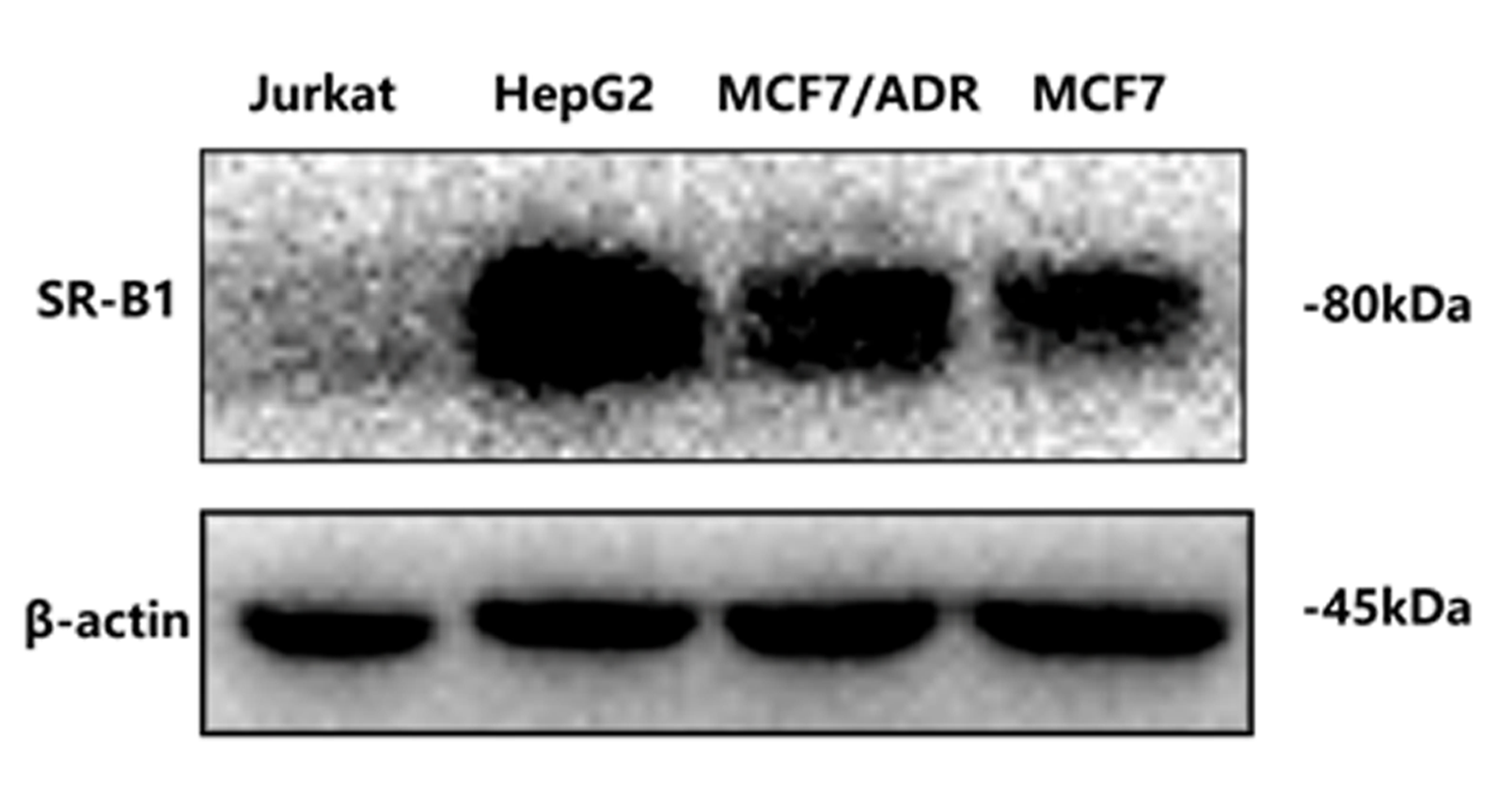

Supplement: Supplementary file 1 [file molecules-26-01280-s001.zip › Supplementary materials/Figure S1.tif]

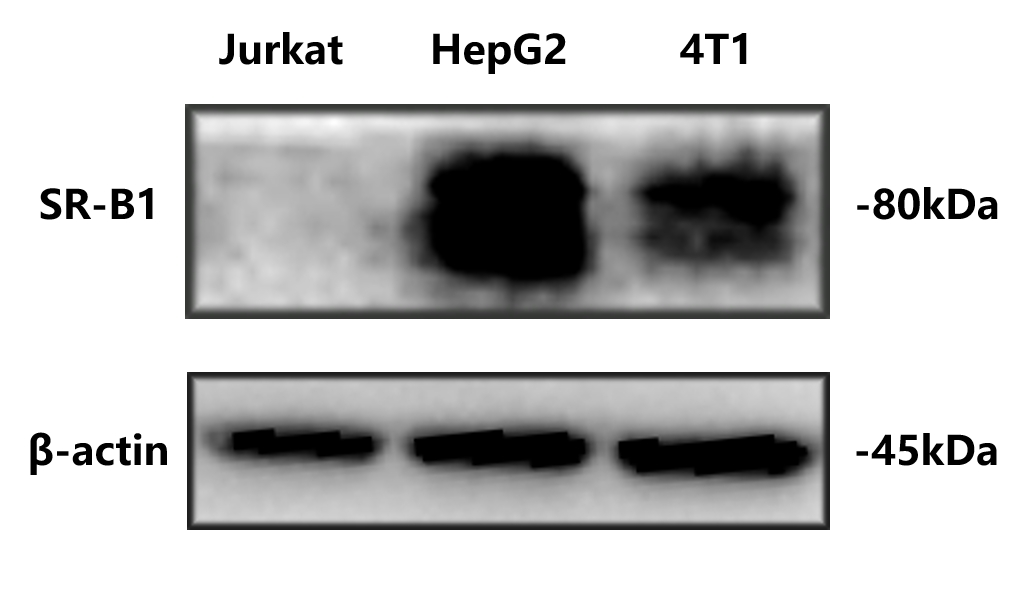

Supplement: Supplementary file 1 [file molecules-26-01280-s001.zip › Supplementary materials/Figure S2.tif]

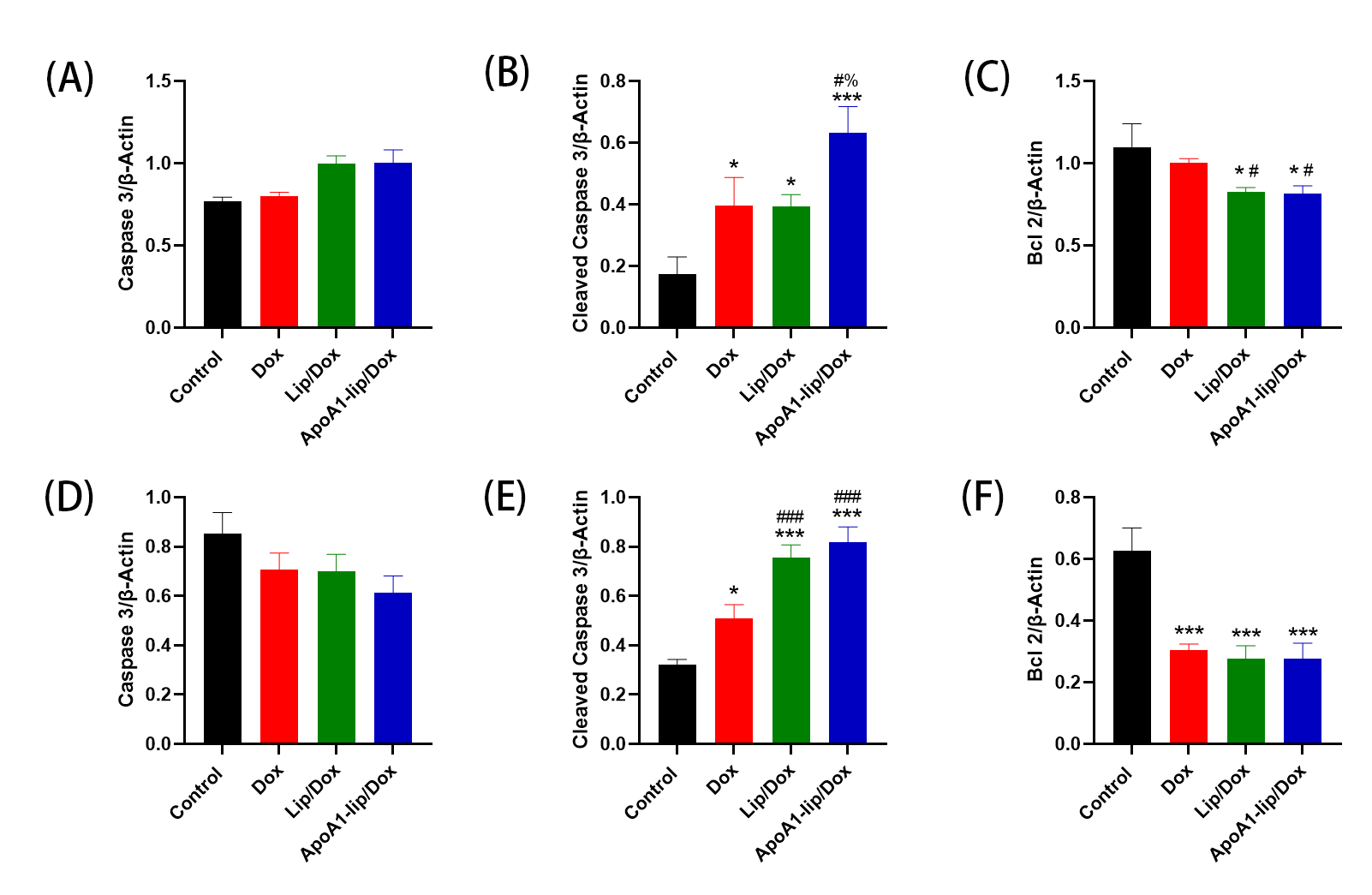

Supplement: Supplementary file 1 [file molecules-26-01280-s001.zip › Supplementary materials/Figure S3.tif]
